# How does competition among wild type mosquitoes influence the performance of *Aedes aegypti* and dissemination of *Wolbachia pipientis*?

**DOI:** 10.1371/journal.pntd.0005947

**Published:** 2017-10-09

**Authors:** Suellen de Oliveira, Daniel Antunes Maciel Villela, Fernando Braga Stehling Dias, Luciano Andrade Moreira, Rafael Maciel de Freitas

**Affiliations:** 1 Fundação Oswaldo Cruz, Fiocruz-RJ, Instituto Oswaldo Cruz, Laboratório de Mosquitos Transmissores de Hematozoários, Rio de Janeiro, Brazil; 2 Fundação Oswaldo Cruz, Fiocruz-RJ, Programa de Computação Científica, Rio de Janeiro, Brazil; 3 Fundação Oswaldo Cruz, Fiocruz-CE, Rio de Janeiro, Brazil; 4 Fundação Oswaldo Cruz, Fiocruz-MG, Instituto René Rachou, Mosquitos vetores: Endossimbiontes e Interação Patógeno-Vetor, Rio de Janeiro, Brazil; University of Wisconsin Madison, UNITED STATES

## Abstract

**Background:**

*Wolbachia* has been deployed in several countries to reduce transmission of dengue, Zika and chikungunya viruses. During releases, *Wolbachia*-infected females are likely to lay their eggs in local available breeding sites, which might already be colonized by local *Aedes* sp. mosquitoes. Therefore, there is an urgent need to estimate the deleterious effects of intra and interspecific larval competition on mosquito life history traits, especially on the duration of larval development time, larval mortality and adult size.

**Methodology/principal findings:**

Three different mosquito populations were used: *Ae*. *aegypti* infected with *Wolbachia* (*w*MelBr strain), wild *Ae*. *aegypti* and wild *Ae*. *albopictus*. A total of 21 treatments explored intra and interspecific larval competition with varying larval densities, species proportions and food levels. Each treatment had eight replicates with two distinct food levels: 0.25 or 0.50 g of Chitosan and fallen avocado leaves. Overall, overcrowding reduced fitness correlates of the three populations. *Ae*. *albopictus* larvae presented lower larval mortality, shorter development time to adult and smaller wing sizes than *Ae*. *aegypti*. The presence of *Wolbachia* had a slight positive effect on larval biology, since infected individuals had higher survivorship than uninfected *Ae*. *aegypti* larvae.

**Conclusions/significance:**

In all treatments, *Ae*. *albopictus* outperformed both wild *Ae*. *aegypti* and the *Wolbachia*-infected group in larval competition, irrespective of larval density and the amount of food resources. The major force that can slow down *Wolbachia* invasion is the population density of wild mosquitoes. Given that *Ae*. *aegypti* currently dominates in Rio, in comparison with *Ae*. *albopictus* frequency, additional attention must be given to the population density of *Ae*. *aegypti* during releases to increase the likelihood of *Wolbachia* invasion.

## Author summary

Several countries are seeking new vector control tools to reduce the transmission of arboviruses such as dengue, chikungunya and Zika. One of these innovative approaches relies on the release of *Aedes aegypti* mosquitoes infected with the endosymbiont *Wolbachia*, since this bacterium can block the aforementioned viruses and interrupt transmission. Countries in Latin America and Southeast Asia have a strong co-occurrence of *Ae*. *aegypti* and *Ae*. *albopictus* in their urban landscapes. Therefore, *Wolbachia*-infected *Ae*. *aegypti* mosquitoes are likely to lay their eggs in local breeding sites already colonized by wild uninfected conspecifics and/or *Ae*. *albopictus* larvae. We conducted experiments to study larval competition with varying larval densities, species proportions and food levels. Interestingly, *Ae*. *albopictus* proved to be a superior competitor under different scenarios: its larvae had superior survivorship, faster development rate and a higher performance index than *Ae*. *aegypti* (both infected and uninfected groups). The presence of *Wolbachia* increased larval survivorship of *Ae*. *aegypti*. Our data show that the population density of wild mosquitoes, especially interspecific competition, can become an additional force to reduce the pace of *Wolbachia* invasion in endemic regions.

## Introduction

Infectious diseases caused by arboviruses are a growing global health concern. Among the disease vectors, mosquitoes from the genus *Aedes* and mostly important *Ae*. *aegypti* (Linnaeus, 1762) and *Ae*. *albopictus* (Skuse, 1894) have a prominent role in transmitting several arboviruses to humans. In the last 50 years, dengue virus (DENV) has shown a 30-fold increase in global incidence, with around 400 million new infections yearly [1–3]. In the last decade, chikungunya became pandemic after spreading from limited regions of Africa and Asia and arriving into the Americas. Two CHIKV genotypes were detected in Brazil: The Asian genotype has probably invaded the country through the Caribbean and the East-Central-South African (ECSA) genotype was first detected in the Bahia State [4,5]. Most recently, Zika virus (ZIKV) emerged in the Pacific and later in the Americas, causing a public health emergency due to its association with microcephaly in newborns [6–8].

The *Ae*. *aegypti* mosquito is more frequently observed in highly urbanized areas. It is extremely well adapted to live in close association with human dwellings. Females blood feed preferentially on humans and lay eggs on man-made containers often located on the surroundings of residences [9–12]. Conversely, *Ae*. *albopictus* is more frequently collected in wooded areas next to humans and tends to bite on a variety of vertebrates outdoors [13]. Both species overlap their spatial distribution in suburban areas, especially in those districts with high vegetation coverage [11,14–16]. Thus, eventually, *Ae*. *aegypti* and *Ae*. *albopictus* share the use of the same breeding sites, which triggers a series of ecological interactions due to the limited resources available.

Many studies have investigated the negative outcomes of competing environment on adult life history traits. Inadequate nutrition during the larval stage of mosquitoes can be associated with reduced wing size, shorter longevity and flight performance, higher susceptibility to arboviral infections and replication under laboratory conditions [17–21]. Density-dependent competition in larval stages causes mortality and thus reduced recruitment to the adult stage, showing that *Ae*. *aegypti* vectorial capacity is strongly dependent on the larval habitat quality.

Effective vector control activities are used as the primary approach to mitigate arbovirus transmission, especially in the absence of vaccines. *Ae*. *aegypti* control still relies massively on source reduction and on using chemicals such as insecticides. However, maintaining infestation level below a theoretical threshold to avoid outbreaks requires a constant and somehow utopic military discipline of field health agents over time [22]. Moreover, the overuse of insecticides favors the dissemination of alleles that confer resistance among wild *Ae*. *aegypti* populations, jeopardizing insecticide efficiency as tools for vector control [23,24]. Due to the low capacity of traditional control measures to reduce mosquito populations, new approaches to mitigate transmission must be tested.

One of the innovative approaches currently being tested is the deployment of the maternally inherited endosymbiont *Wolbachia pipientis* into wild mosquito populations [25]. The use of *Wolbachia* as a natural control agent is supported by findings showing that *Ae*. *aegypti* females infected with the *w*Mel strain are able to block DENV, CHIKV and ZIKV [26–28]. Accordingly, *Wolbachia* releases may be used to mitigate arbovirus transmission by two different strategies: suppression of mosquito population by massive male-releases or the substitution of a highly susceptible population by one that blocks arbovirus transmission [29,30].

*Wolbachia* deployments are taking place in five countries, including Brazil (www.eliminatedengue.com). When *Wolbachia*-infected mosquitoes are released, females tend to behave as their wild counterparts, i.e., will blood feed on local householders and lay eggs in the available breeding sites. Considering that in Rio de Janeiro city there is a strong co-occurrence of *Ae*. *aegypti* and *Ae*. *albopictus* [11, 16], females carrying *Wolbachia* will eventually lay eggs in containers already colonized by wild *Aedes* sp. mosquitoes. Therefore, our main objectives were to estimate the deleterious effects of larval competition on mosquito life history traits, but also to determine to what extent larval competition of wMel-*Ae*. *aegypti* mosquitoes with wild *Ae*. *aegypti* and *Ae*. *albopictus* may jeopardize *Wolbachia* invasion.

## Materials and methods

### Mosquitoes

We used three different mosquito populations: *Ae*. *aegypti* infected with *Wolbachia* (*w*MelBr strain), wild *Ae*. *aegypti* and wild *Ae*. *albopictus*.

### *Ae*. *aegypti* infected with *Wolbachia* strain *w*MelBr

The lineage of *Ae*. *aegypti* with *w*Mel was imported from Australia to Brazil (IBAMA license 11BR005873/DF). Briefly, a backcrossing with 250 virgin females (*w*Mel) and 200 wild males was conducted for nine consecutive generations, producing *w*MelBr [31]. After that period, the *w*MelBr colony was outcrossed every five generations with 10% wild males from a pool of four districts (Jurujuba, Tubiacanga, Urca and Vila Valqueire) with high nuclear genome homogeneity across Rio to refresh the genetic pool [32]. We used the F19 of *w*MelBr generation.

### *Ae*. *aegypti* and *Ae*. *albopictus* wild population

Wild *Ae*. *aegypti* and *Ae*. *albopictus* mosquitoes were obtained from four districts in Rio de Janeiro city (the same districts with which the *w*MelBr colony was outcrossed) by collecting eggs laid on the wooden paddle of ovitraps. A total of thirty ovitraps were installed uniformly in each area, of approximately 1 km^2^, to represent the genetic variation of the wild population. Wooden paddles were brought to Fiocruz, eggs were hatched and larvae were classified using taxonomic keys [33]. Larvae of each species were pooled and reared in dechlorinated water and fed with TetraMin (fish food), maintained in a climate controlled insectary, at 26 ± 1°C and 70 ± 10% relative humidity. Adult females were kept under a 12:12 hour light:dark cycle, *ad libitum* access to sugar solution (10%) and blood fed twice a week using anesthetized mice (CEUA L-0007/09). Eggs were stored under insectary conditions until the experiment. We used the F1 of wild *Ae*. *aegypti* and F2 of *Ae*. *albopictus*.

### Semi-field conditions

The larval competition experiments were performed in a semi-field setting, an open building located at the Army Institute of Biology in Rio de Janeiro, Brazil (22°53’34”S, 43°14’33”W), but with limited control access to unauthorized personnel. The experiment was subject to the influences of climate variation, such as humidity and air temperature, as well as rainfall. These conditions were continuously recorded by means of a weather station (Instrutemp, ITWH model 1080) installed on site.

### Experimental design

The intraspecific larval competition of *Ae*. *aegypti* (with and without *w*MelBr) and interspecific competition with *Ae*. *albopictus* were investigated by monitoring the development of larvae at different densities, species proportions, and food levels in containers. Twenty-one treatments were set and used different proportions of wild *Ae*. *aegypti*: *w*MelBr*-Ae*. *aegypti*: *Ae*. *albopictus* (20:0:0, 40:0:0, 60:0:0, 0:20:0, 0:40:0, 0:60:0, 0:0:20; 0:0:40, 0:0:60, 20:0:20, 30:0:30, 20:0:40, 40:0:20, 0:20:20, 0:30:30, 0:20:40, 0:40:20, 20:20:0, 30:30:0, 20:40:0, 40:20:0). The densities evaluated herein were based on that of Braks et al. (2004) [19] and represent larval crowding in nature [12,34]. Larvae were placed as L1 in 400 ml of tap water into black plastic containers (9.5 cm in height, 8.5 cm base diameter). Each treatment had eight containers with two distinct food levels. The food consisted of 0.25 or 0.50 g of Chitosan (an analogue of insect chitin used to simulate the remains of arthropods), and fallen avocado leaves (extra source of natural nutrition commonly used in *Aedes* competition assays), in the same proportion, that were collected, washed, dried, broken into small pieces and weighed. Therefore, our experimental design consisted of 200 plastic containers (12 cm in diameter x 15 cm in height).

Each container was identified and received the appropriate quantity of Chitosan and leaf litter, with 400 ml of tap water, three days before the addition of larvae. Containers were covered with black tulle to prevent oviposition by wild mosquitoes. One hour after eggs were hatched, larvae were counted with the help of a stereo microscope and then added to their appropriate containers. Each container was monitored daily for the presence of pupae, which were collected and placed in small covered vials (6.5 cm height x 2.5 cm diameter) and kept until adult emergence. On the day of emergence, adults were killed with acetyl acetate and, after being sexed, one wing was removed. Wing length was defined as the distance from the axillary incision to the apical margin excluding the fringe [35]. The experiment ended when the last pupa became adult.

### Detection of *w*MelBr through molecular assays

In treatments with the presence of *w*MelBr and wild *Ae*. *aegypti* simultaneously, all adults and dead pupae were screened for *Wolbachia*. Screening was performed using the Taqma^n^ multiplex Real Time—Polymerase Chain Reaction. Adult mosquitoes and dead pupae were individually screened on ViiA7 Real Time PCR machine (Life Technologies). The genomic DNA was extracted using a squash buffer (0.1 M NaCl; 10 mM Tris Base; 1 mM EDTA; pH 8.2) supplemented with 9 μg of Proteinase K per mosquito (Qiagen). After macerating the mosquitoes with a 2mm glass-bead on a Mini-beadbeater (Biospec Products), samples were placed on a thermocycler following the thermal cycle: 56° C for 5 minutes and 98° C for 15 minutes. Genomic DNA was diluted 1:10 in ultra-pure water and then used as the template for *Wolbachia* screening. We used the WD0513 gene that amplifies a fragment of 110 bp with the following primers: TM513-Forw: CAA ATT GCT CTT GTC CTG TGG and TM513-Rev: GGG TGT TAA GCA GAG TTA CGG and TM513-probe 5’-/FAM Cy5/ TGA AAT GGA AAA ATT GGC GAG GTG TAG G -–BHQ-1/-3’. In the same reaction, a ribosomal gene from *Ae*. *aegypti* that amplifies a fragment of 68 bp was analyzed with the primers: RPS17-Forw: RPS17-Forw: 5’- TCC GTG GTA TCT CCA TCA AGC T- 3’ and RPS17-Rev: 5’- CAC TTC CGG CAC GTA GTT GTC- 3’, and RPS17-probe: 5’-/FAM/CAG GAG GAG GAA CGT GAG CGC AG/3BHQ_1/-3’. Negative and positive controls of *Ae*. *aegypti* (with and without *w*MelBr) and *Ae*. *albopictus* were used in all reactions.

Reagents used in the qPCR were: 5 μL of TaqMan Universal PCR Master Mix (Thermo Fisher), 0.5mM of RPS17 primers, 0.6mM of TM513 primers, 0.1mM of RPS17 probe, 0.25mM of TM513 probe and 1μL of diluted DNA. Water was added to complete a final volume of 10 μL.

### Data analyses

Three biological aspects were observed throughout the experiment: larval survivorship, developmental time and wing length. Survivorship was calculated, for each container, by the frequency of larvae that reached the adult stage. Developmental time per container was calculated as the average number of days from hatching until the emergence of the adult was observed in the plastic vial.

An important parameter in population ecology is the performance index *λ’*, related to the growth rate *r’* by *λ*’ = exp(*r*^´^). We calculate *λ’* using values of observed biological aspects, such as survival of immature, development time and adult size of cohorts of mosquitoes, for each replicate. An estimate of the performance index has been adapted by [36] from the equation established by [37] using *r’* as a measurement of population growth. According to this index, the condition *λ*′ > 1.0 represents an increase in the population, whereas condition *λ*′ < 1.0 points to a population decrease. The *λ’* index was calculated for each replicate as follows:

$$r^{\prime }=\frac{ln(\frac{1}{N_{0}}\sum_{x}A_{x}f({\bar{w}}_{x}))}{D+\frac{\sum_{x}xA_{x}f({\bar{w}}_{x})}{\sum_{x}A_{x}f({\bar{w}}_{x})}},$$

where *N*_*0*_ is the initial number of females in a cohort, which we assumed to be 50% of the added larvae, since the sex ratio of the species studied here is generally 1:1 [38,39]; *A*_*x*_ is the number of adult females on day *x*; *w*_*x*_ is the average size of the female wing on day *x*; fecundity of females is modeled by a function *ƒ (w*_*x*_*)* of the wing size, as proposed for *Ae*. *aegypti* [40] and *Ae*. *albopictus* [41]. No significant differences in fecundity have been found due to *Wolbachia* infection [31], thus we assumed the same relationship between mosquito size and fecundity for infected and uninfected mosquitoes. *D* is the time required (in days) for a newly hatched female to mate, blood feed and lay eggs. In our experiments, *D* is typically equal to the number of days that a female takes to reach the adult stage plus four days, the length of the first gonotrophic cycle [42].

The effects of competition conditions on the performance of *Ae*. *aegypti* infected with *w*MelBr were analyzed using a Generalized Linear Model (GLM). Development time, wing length, survival proportion and the performance index were analyzed each separately as outcomes using as explanatory variables: species, nutrients, competing numbers of wild *Ae*. *aegypti*, *Ae*. *albopictus*, and *Ae*. *aegypti* with *w*MelBr. For development time, performance index, and wing length we used a normal distribution and logarithmic link function. For survivorship we used logistic regression models with a binomial family/logit link function. For each of the outcomes we selected the model with lowest Akaike Information Criterion (AIC). P-values lower than 0.05 were considered significant. We used R 3.0.1 software for these analyzes.

The index values of *λ*' were used to make a model to simulate the impact of different levels of infestation of *Ae*. *aegypti* wild type and *Ae*. *albopictus* in the performance of *Ae*. *aegypti* with *w*Mel*Br* in larval competition. Three nonlinear regressions were applied to each of the indices *λ*' for the three populations: wild *Ae*. *aegypti*, *Ae*. *albopictus* and *Ae*. *aegypti* with *w*MelBr, with the number (*n*_*x*_) of individuals in each cohort *x* (aeg for wild *Ae*. *aegypti*, albo for *Ae*. *albopictus*, wmel for *Ae*. *aegypti* with *w*MelBr) in competition, according to the following model: *log (λ’) ~ n*_*aeg*_ *+ n*_*albo*_ *+ n*_*wmel*_. These analyses allowed us to evaluate the effect on the performance index when increasing both interspecific and intraspecific competition. The values obtained in the regressions were used to simulate the interspecific competition among the three populations.

Once coefficients for interspecific competition were obtained we evaluated the intensity of interspecific competition that makes the growth rate negative, i.e., r´ = *log (λ’) < 0*. For instance, if population of *Ae*. *aegypti* with *w*MelBr suppresses the wild *Ae*. *aegypti* population, this permitted us to evaluate the frequency of *Ae*. *albopictus* that causes a severe interspecific competition that might compromise sustained growth of *Ae*. *aegypti* with *w*Mel. In this case, we find value for r´_wMel_ = *log (λ’*_wMel_*) = β*_*wmel*_ *+ α*_*wmel*_ *n*_*albo*_ *+ γ*_*wmel*_ *n*_*wmel*_ *< 0*, where *β*, *α*_*wmel*_, and *γ*_*wmel*_ are coefficients obtained in the regression analysis.

### Ethical issues

The use of anesthetized mice to blood feed mosquitoes was authorized by Fiocruz Ethical Committee for Animal Use (CEUA L-0007/09), which follows the National guidelines for the scientific use of animals disposed on the Law 11.794/2008.

## Results

### Survivorship

**Fig 1:**
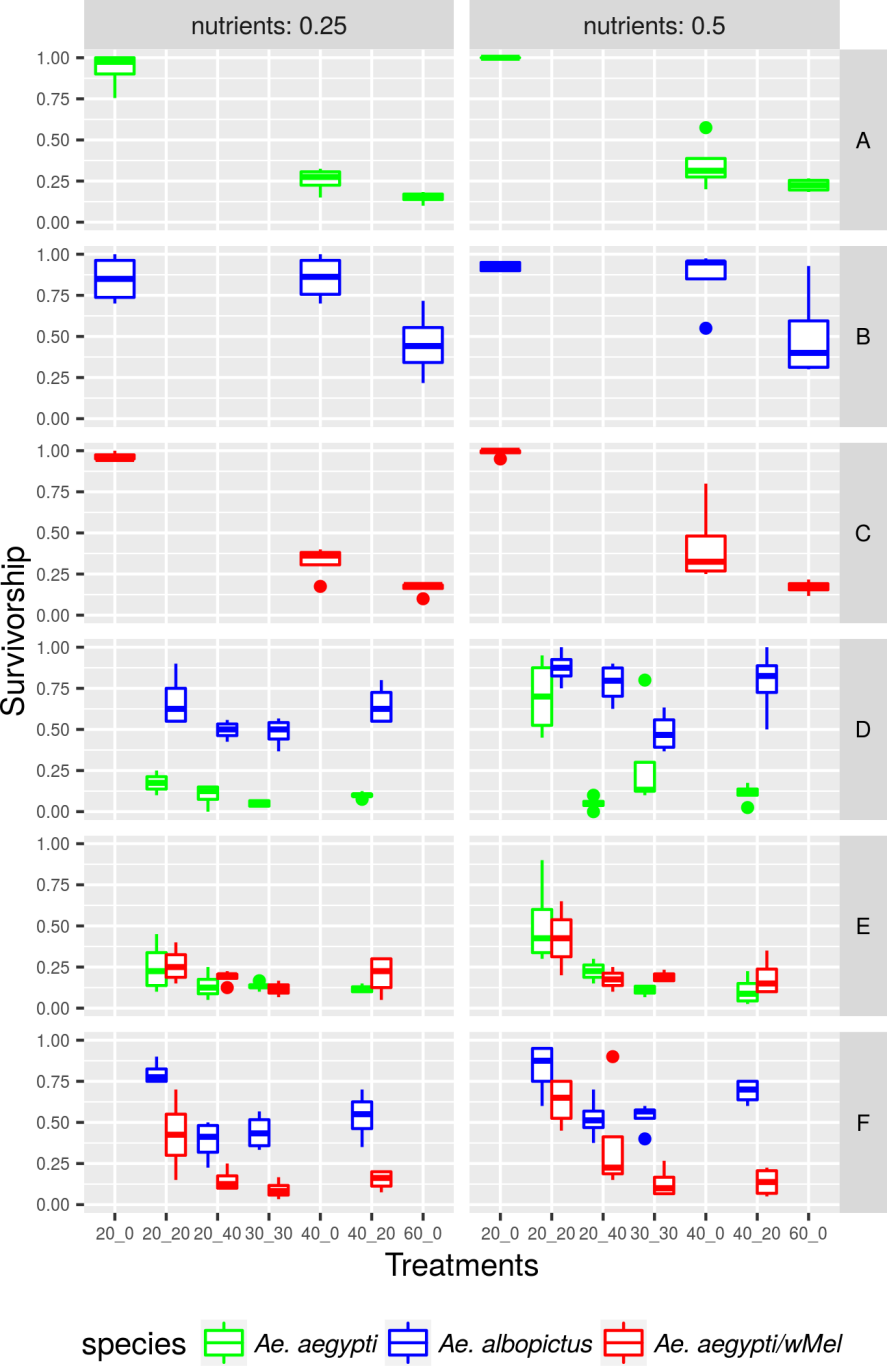
Variation of wild *Aedes aegypti*, *Ae*. *aegypti* with *wMelBr* and *Ae*. *albopictus* survivorship according to the amount of resources and treatment in which each population was reared. A. Intraspecific competition among *Ae*. *aegypti* larvae B. Intraspecific competition among *Ae*. *albopictus* larvae. C. Intraspecific competition among *Ae*. *aegypti/w*Mel larvae. D. Interspecific competition between *Ae*. *aegypti* and *Ae*. *albopictus*. E. Interspecific competition between *Ae*. *aegypti* and *Ae*. *aegypti/w*Mel. F. Interspecific competition between *Ae*. *aegypti/w*Mel and *Ae*. *albopictus*.

Under intraspecific competition, survival was inversely proportional to larval density in the three tested populations, as expected (*Ae*. *albopictus*: t = -27.2, P<0.05, *Ae*. *aegypti*: t = -28.3, P<0.05, *Ae*. *aegypti* with *w*MelBr: t = -26.3, P<0.05). *Ae*. *albopictus* presented higher tolerance for increasing competition than wild *Ae*. *aegypti* and *Ae*. *aegypti* with *w*MelBr. On the other hand, *Ae*. *aegypti* presented a significant decrease in survivorship when larval density per container doubled. This pattern was observed independently of *Wolbachia* presence (Fig 1, Table 1).

**Table 1:** Generalized Linear Model to determine the influence of the number of larvae, mosquito population, nutrients and its interactions on the larvae survivorship. The model selected presented the lowest Akaike Information Criterion (AIC), which is informed in Table.

Variables	Coefficient estimate	Confidence Interval	p-value
Number of *Ae*. *albopictus*	-0.080	(-0.085, -0.074)	< 0.001
Number of *Ae*. *aegypti/w*Mel	-0.082	(-0.088, -0.076)	< 0.001
Number of wild *Ae*. *aegypti*	-0.075	(-0.080, -0.069)	< 0.001
*Ae*. *albopictus* compared to wild *Ae*. *aegypti*	2.325	(1.886, 2.276)	< 0.001
*Ae*. *aegypti/w*Mel compared to wild *Ae*. *aegypti*	0.853	(0.389, 1.318)	< 0.001
Nutrients–*Ae aegypti*	2.444	(1.651, 3.238)	< 0.001
Nutrients–*Ae*. *albopictus*	1.963	(1.301, 2.625)	< 0.001
Nutrients–*Ae*. *aegypti/w*Mel	1.217	(0.455, 1.978)	< 0.001
AIC	1814			

Under interspecific competition, the survival of *Ae*. *albopictus* and *Ae*. *aegypti* with *w*MelBr larvae was significantly higher than survival of wild *Ae*. *aegypti*, irrespective of the amount of food resources (Table 1). Nonetheless, competitive advantage of *Ae*. *albopictus* over wild *Ae*. *aegypti* seemed slightly more evident in the most stressful and competitive treatments. *Ae*. *aegypti* with *w*MelBr also survived less than *Ae*. *albopictus*, although its survival is marginally higher than that observed for wild *Ae*. *aegypti*. In some particular treatments, *Ae*. *aegypti* larvae infected with *w*MelBr presented better survival than wild *Ae*. *aegypti*.

### Developmental time

**Fig 2:**
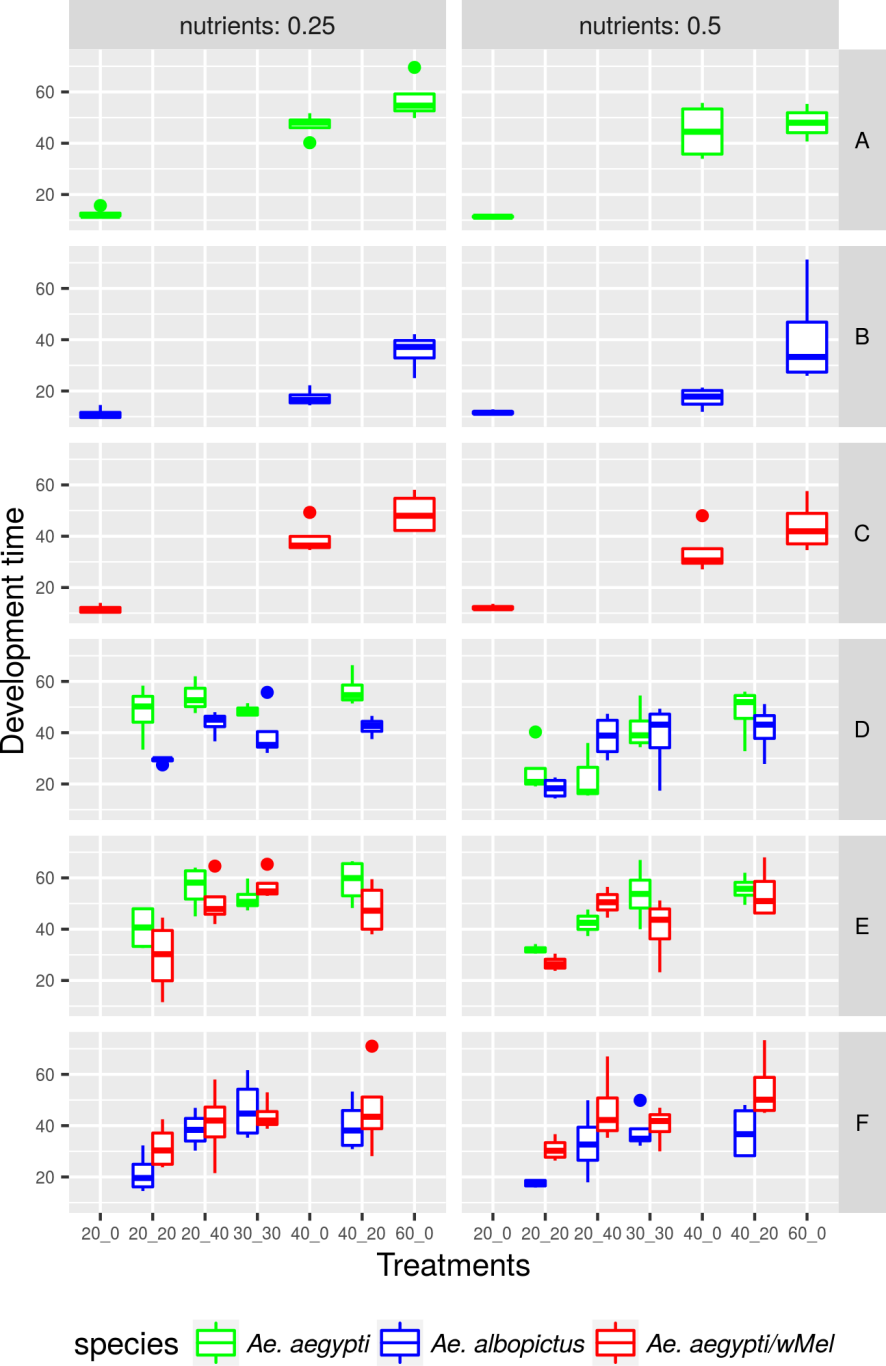
Variation of wild *Aedes aegypi*, *Ae*. *aegypti* with *w*MelBr and *Ae*. *albopictus* development time from L1 to adult according to the amount of resources and treatment in which each population was reared.

Under intraspecific competition, overcrowding was directly related to the increase in developmental time (DT) (P<0.05). Wild *Ae*. *aegypti* and those infected with *w*MelBr have a longer DT starting at 40 larvae per container, while *Ae*. *albopictus* DT was notably affected only at a higher density, i.e., 60 larvae per container (Fig 2, Table 2). Wild *Ae*. *aegypti* presented the longest DT at high densities with an average duration of 57.17 and 47.99 days in low and high food resources, respectively. Under interspecific competition, *Ae*. *aegypti* with *w*MelBr had a similar DT to *Ae*. *albopictus* at high food resources, but was outcompeted when food resources were scarce. Interestingly, *Ae*. *albopictus* presented a shorter DT than wild *Ae*. *aegypti* (t = 4.33, P<0.05), but the presence of *Wolbachia* did not alter the DT in *Ae*. *aegypti*.

**Table 2:** Generalized Linear Model to determine the influence of the number of larvae, mosquito population, nutrients and their interactions on the larvae development time (DT) from L1 to adult. The model selected presented the lowest Akaike Information Criterion (AIC).

Variables	Coefficient estimate	Confidence Interval	p-value
Number of *Ae*. *albopictus*	0.021	(0.017, 0.025)	< 0.001
Number of *Ae*. *aegypti/w*Mel	0.024	(0.021, 0.028)	< 0.001
Number of wild *Ae*. *aegypti*	0.025	(0.022, 0.029)	< 0.001
Nutrients	-0.436	(-0.678,-0.195)	< 0.001
Species/Pop.–*Ae*. *albopictus*	-0.209	(-0.304, -0.115)	< 0.001
Species/Pop.–*Ae*. *aegypti/w*Mel	-0.068	(-0.153, 0.018)	0.121
AIC	1921			

### Female wing size

**Fig 3:**
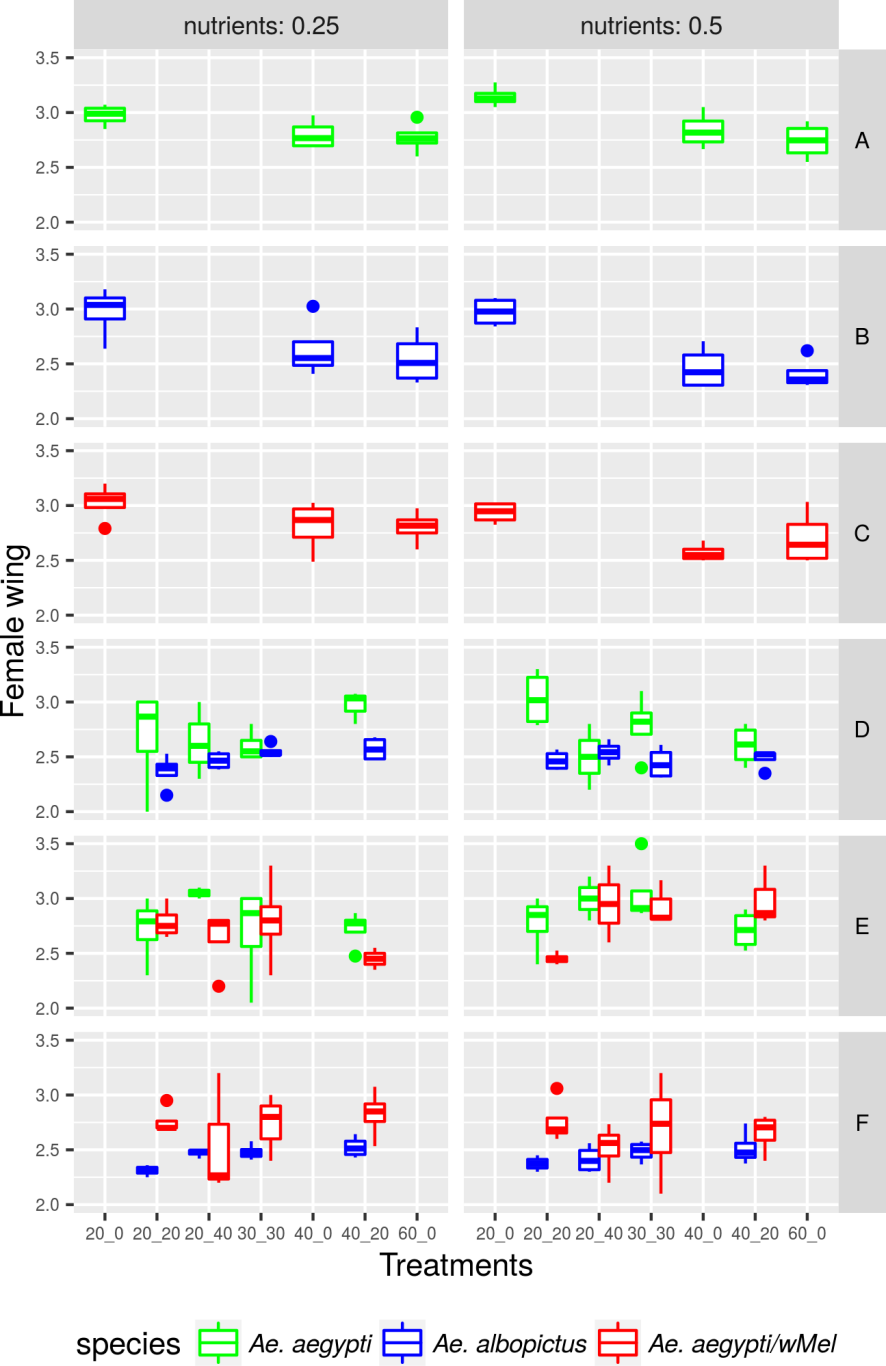
Variation of wild *Aedes aegypi*, *Ae*. *aegypti* with *w*MelBr and *Ae*. *albopictus* female wing size according to the amount of resources and treatment in which each population was reared.

The three populations had a significant decrease in wing size due to overcrowding (Table 3, Fig 3). *Ae*. *albopictus* had a sharper decrease than wild *Ae*. *aegypti* and *Ae*. *aegypti* with *w*MelBr. The presence of *Wolbachia* did not seem to influence mosquito wing size. The amount of nutrients had a positive effect for wild *Ae*. *aegypti* and a negative effect for *Ae*. *albopictus*.

**Table 3:** Generalized Linear Model to determine the influence of the number of larvae, mosquito population, nutrients and their interactions on the wing size of adult females. The model selected presented the lowest Akaike Information Criterion (AIC).

Variables	Coefficient estimate	Confidence Interval	p-value
Number of *Ae*. *albopictus*	-0.003	(-0.004, -0.002)	< 0.001
Number of *Ae*. *aegypti/w*Mel	-0.002	(-0.003, -0.001)	0.003
Number of wild *Ae*. *aegypti*	-0.002	(-0.003, -0.001)	< 0.001
Nutrients–*Ae*. *aegypti*	0.109	(0.009, 0.209)	0.034
Nutrients–*Ae*. *albopictus*	-0.122	(-0.225, -0.019)	0.022
Nutrients–*Ae*. *aegypti/w*Mel	0.016	(-0.085, 0.117)	0.752
AIC	2.29			

### Performance index (*λ*’)

**Fig 4:**
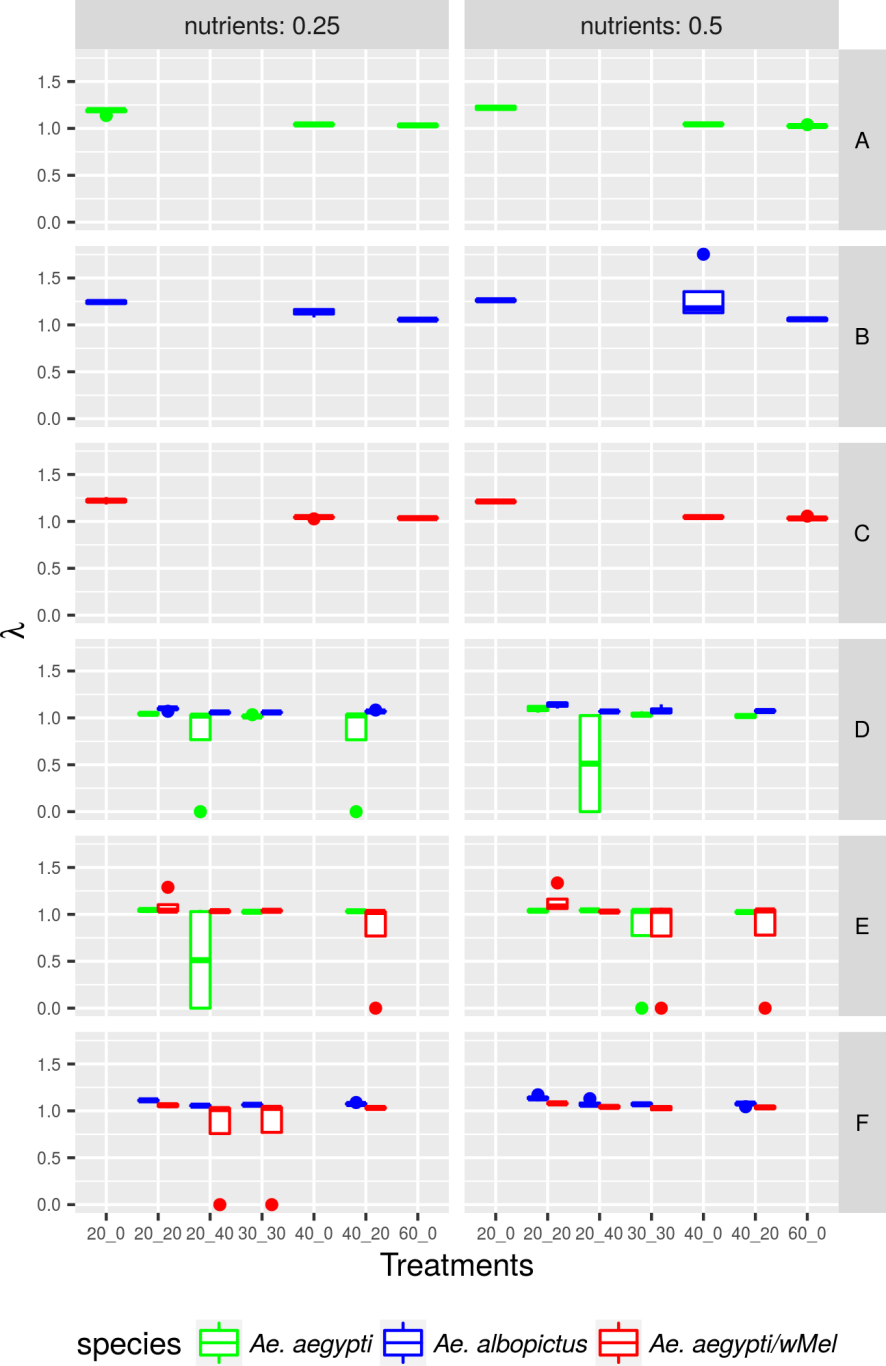
Variation of wild *Aedes aegypi*, *Ae*. *aegypti* with *wMelBr* and *Ae*. *albopictus* performance index according to the amount of resources and treatment in which each population was reared.

Overall, overcrowding had a significant effect on the performance of the three populations (Fig 4, Table 4). The value of *λ*' for wild *Ae*. *aegypti* and *Ae*. *aegypti* with *w*MelBr suffered a reduction from 1.2 to 1.0 when the larval density was doubled (treatment 2). On the other hand, the *λ*' for *Ae*. *albopictus* was only reduced to 1.0 when the larval density was tripled (treatment 3). Remarkably, all populations tested were able to maintain *λ*' above 1 under low densities, meaning that they could be successfully sustained in the wild. Under these experimental settings, *Ae*. *albopictus* showed superior performance to wild *Ae*. *aegypti* and the presence of *Wolbachia* did not seem to affect *Ae*. *aegypti* performance (Fig 4, Table 4).

**Table 4:** Generalized Linear Model to determine the influence of the number of larvae, mosquito population, nutrients and its interactions on the composite performance index (λ’). The model selected presented the lowest Akaike Information Criterion (AIC).

Variables	Coefficient estimate	Confidence Interval	p-value
Number of *Ae*. *albopictus*	-0.007	(-0.009, -0.005)	< 0.001
Number of *Ae*. *aegypti/w*Mel	-0.006	(-0.009, -0.004)	< 0.001
Number of wild *Ae*. *aegypti*	-0.006	(-0.008, -0.004)	< 0.001
*Ae*. *albopictus*	0.164	(0.083, 0.246)	< 0.001
*Ae*. *aegypti*/*wMel*	0.039	(-0.043, 0.122)	0.351
AIC	1814			

### Effects of competition intensity on the performance index (λ') of competing species

**Fig 5:**
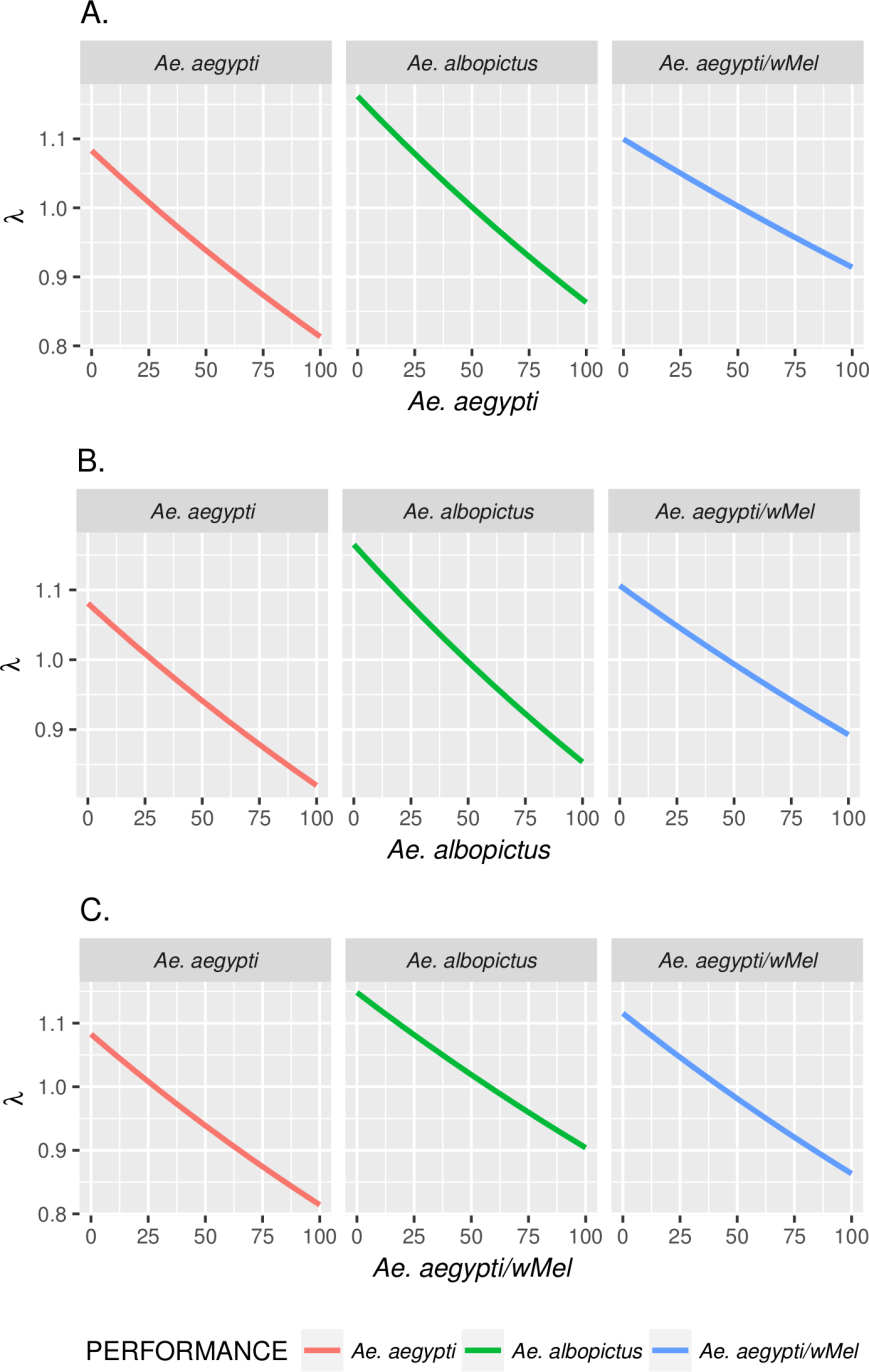
How the performance index varies in scenarios with both intraspecific and interspecific competition among the three populations. Performance index λ for *Ae*. *aegypti* (red), *Ae*. *albopictus* (green), *Ae*. *aegypti/w*Mel (blue) populations when increasing (A) *Ae*. *aegypti* competition (B) *Ae*. *albopictus* competition (C) *Ae*. *aegypti/w*Mel competition. When varying the number of larvae of a population, the other two populations are at fixed levels (n = 20).

We simulated interspecific competition among the three different populations applying the results from the nonlinear regression analyzes for each of the *Ae*. *aegypti*, *Ae*. *albopictus*, *Ae*. *aegypti* with *w*Mel performance indices. As expected, increasing either intraspecific or interspecific competition makes the performance index smaller for the three populations. In Fig 5, when performance indices reach values below lines for λ = 1, the interspecific competition does not allow a positive growth rate. We generally observe that the values under which *Ae*. *albopictus* can sustain a positive growth rate are larger than values for both *Ae*. *aegypti* and *Ae*. *aegypti* with *w*Mel populations.

**Fig 6:**
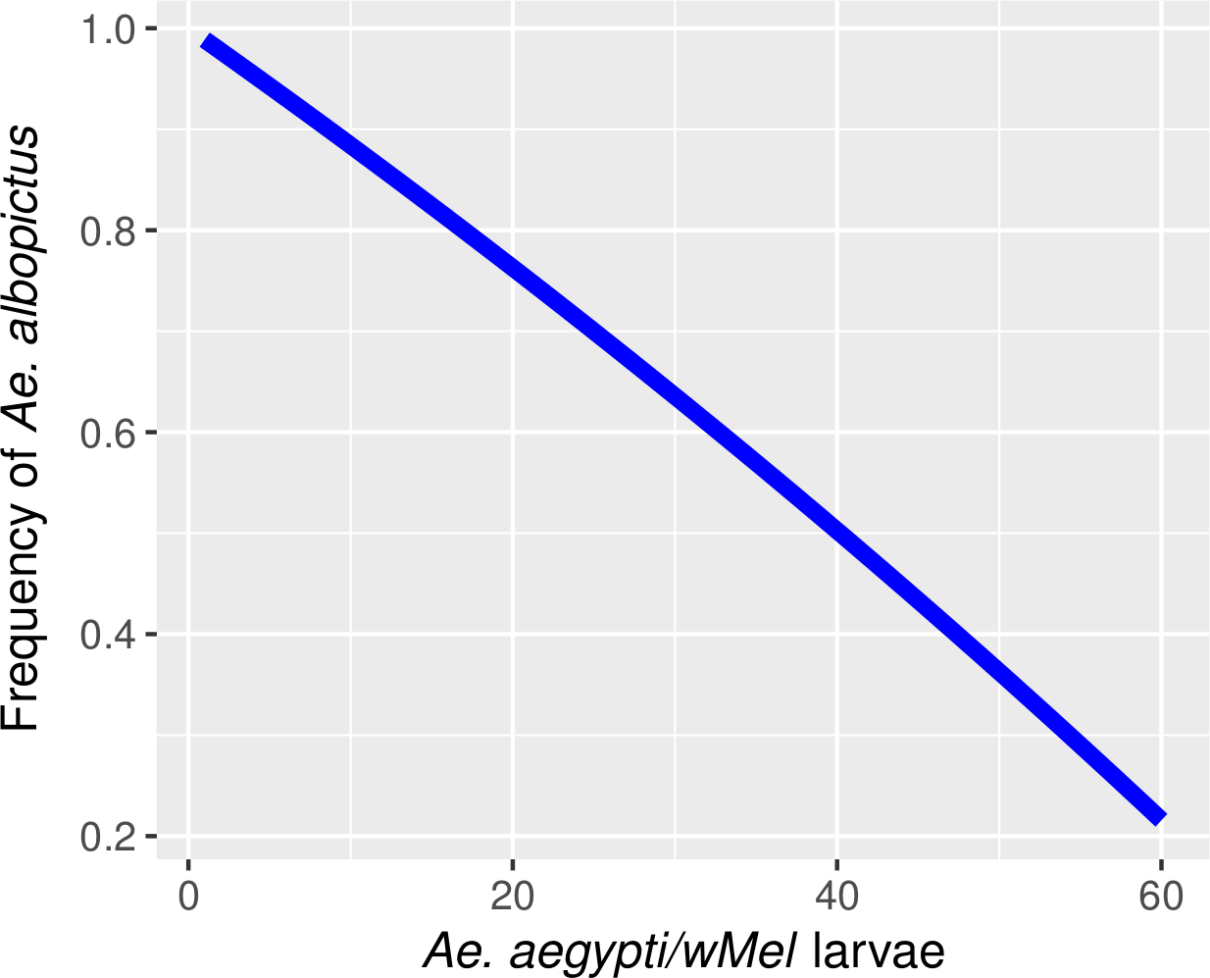
Frequency of *Ae albopictus* required for negative growth rate of *Ae*. *aegypti/w*Mel, when fixing the intraspecific competition at different numbers of larvae (x-axis).

We also studied the frequency of *Ae*. *albopictus* that could make interspecific competition intense enough to make *Ae*. *aegypti*/*w*Mel performance index *λ*_wmel_<1, i.e. impacting severely on the sustained growth assuming only larval competition (Fig 6). As the number of *Ae*. *aegypti*/*w*Mel larvae increases (intraspecific competition), the frequency of *Ae*. *albopictus* that causes the performance index to reach an unsustainable level *λ*_wmel_<1 decreases.

## Discussion

The deployment of *Wolbachia* to reduce dengue transmission is currently being undertaken in several regions of the world. During releases, *Ae*. *aegypti* mosquitoes infected with *Wolbachia* will lay eggs in breeding sites in which wild mosquitoes previously colonized, i.e., intra-specific competition with local *Ae*. *aegypti* and other native mosquitoes such as *Ae*. *albopictus* might be an important issue to determine the pace of *Wolbachia* invasion. Due to the co-occurrence of *Ae*. *aegypti* and *Ae*. *albopictus* in several countries of Southeast Asia and Latin America, we investigated how the intra- and interspecific competition with *Ae*. *albopictus* might undermine *w*Mel invasion.

We explored three critical aspects of mosquito biology under different competition scenarios: larval survivorship, developmental time and wing length. Using these estimates, we calculated a performance index that is related to growth rate for wild *Ae*. *aegypti*, *Ae*. *albopictus*, and *Ae*. *aegypti* with *w*MelBr.

Overcrowding significantly reduced larval survivorship in the three populations tested, as expected [18,19,43–45]. Our data show that under intraspecific competition settings, larval survivorship decreased more intensely for *Ae*. *aegypti* than for *Ae*. *albopictus*. Also, the presence of *Wolbachia* did not affect this pattern (Fig 1). The effects of *Ae*. *albopictus* density on *Ae*. *aegypti* mortality and vice-versa has been evaluated elsewhere [19,34,46,47]. In summary, one of these populations gets more severely affected when the density of the other increases, and this increase in mortality might be seen in the larval or adult stage [48,49]. *Ae*. *albopictus* is frequently pointed as a better competitor than *Ae*. *aegypti* [19,34,36,46,50,51] as well as other species such as *Ae*. *japonicus* [52] and *Culex pipiens* [53]. Larvae survival under interspecific competition conditions may vary due to the difference in efficacy with which larvae exploit food resources [54]. *Ae*. *albopictus* showed a larval survivorship higher than that observed for both wild and infected *Ae*. *aegypti*. This finding strongly suggests *Wolbachia* has limited role in affecting larvae mortality under competitive scenarios.

The *w*Mel strain has relatively mild effects on mosquito fitness [25,55], but interestingly, a superior survivorship of infected larvae was reported in a competitive environment when compared with uninfected larvae [39]. On the other hand, *Wolbachia* infection reduced the tolerance of *Ae*. *aegypti* larvae to starvation, probably due to an increasing rate of depletion of accumulated energy reserves [45]. Our data support an overall beneficial impact of *Wolbachia* infection on *Ae*. *aegypti* larval survivorship, since infected larvae present a superior survival rate than their uninfected counterparts (the exception being observed in starvation scenarios, in which *Wolbachia* reduced larval survivorship).

The development time from egg hatch to adult is a critical fitness aspect of mosquito biology under field conditions. Delayed larvae are more subject to external factors such as predation [56], water evaporation and breeding site treatment or removal. Our results show that nutrient levels caused longer development time for all three populations when food resources were scarce [19,48,57]. *Ae*. *albopictus* had a rapid development time when compared with *Ae*. *aegypti* [11] even in the more competitive treatments. The presence of *Wolbachia* did not accelerate *Ae*. *aegypti* developmental time. Differences in larvae development time were highest in treatments with interspecific competition with 0.25 g of litter. The exception was the *Ae*. *albopictus*/*Ae*. *aegypti* with *w*Mel, in which larvae of both populations have distinct development time differences only at 0.50 g litter, with the former developing faster than the latter.

Overall, results regarding the influence of *Wolbachia* on larval development time are conflicting. The experimental design and settings established in our competitive assays were unable to detect any changes on *Ae*. *aegypti* development time due to *Wolbachia* presence. At intermediate (50 larvae/tray) and high densities (250 larvae/tray), *w*Mel infection led to more rapid larval development for both males and females, with no effect under a less crowded and more stressful condition [28]. Opposing results to our data, a slight delay was observed in *w*Mel infected larvae related to their uninfected counterparts [39]. Despite these findings in disagreement, few strains of *Wolbachia* are known to modify adult feeding behavior, and might interfere with larval foraging capability as well [26,56,58,59]. Potential explanations for the effects of *Wolbachia* on mosquito larval development time involve immune up-regulation or increased metabolism observed in the adults, which may also influence larval development rate [26,60]. Other aspects still need an evaluation to better understand the effect of *Wolbachia* on immature development time, such as the effects of the population genetic background, *Wolbachia* strain and experimental design.

Mosquito body size is ultimately a manifestation of larval habitat quality and can produce significant effects on an insect’s fitness and then alter mosquito vectorial capacity [12,61,62]. Physiological stress in juvenile stages produces negative effects that may pass into adulthood [63]. For instance, highly competitive environments produce mosquitoes with a small wing length, which are less likely to promote *Wolbachia* invasion since they should blood feed more often, possess shorter longevity and lower flight performance than bigger mosquitoes [18–21]. Hence, *Ae*. *aegypti* vectorial capacity is strongly dependent on the larval habitat quality [11,17–21]. Previous reports have shown an inversely proportional correlation between wing size and larval density in *Ae*. *aegypti*, as we observed [28,45]. Our results indicate reduction in mosquito size due to overcrowding in all three populations, which is highly expected [19, 49]. *Ae*. *albopictus* wing size was consistently smaller than *Ae*. *aegypti* in almost all treatments, with visible differences when competition was intra or interspecific. The interaction between nutrients and population produced unexpected results. *Ae*. *albopictus* body size decreased at 0.5 g litter when compared with the 0.25 g treatment. Interestingly, body size of *Ae*. *aegypti* with *w*Mel was not affected by availability of food resources. Hence, from the perspective of *Wolbachia* deployment, the infection with *w*Mel strain does not pose a significant disadvantage during competition against wild mosquitoes [39].

We used three population growth correlates, i.e. larval survivorship, time to adulthood and adult wing size to estimate a composite index of mosquito performance (*λ*') for each container [36,37]. Overall, larval density negatively affected the performance index *λ*' of the three populations, but remarkably only *Ae*. *albopictus* population growth was positive in all treatments. In fact, population growth of *Ae*. *albopictus* was significantly superior from the observed for wild *Ae*. *aegypti*, while the presence of *Wolbachia* provided no advantage to infected *Ae*. *aegypti*. Interspecific assays using *Ae*. *aegypti* and *Ae*. *albopictus* at different densities have shown a superior competitive ability of the the latter [19,34,36]. Despite being frequently described as a superior larval competitor to *Ae*. *aegypti*, these two species coexist in much of Brazil and in southeast US and Southeast Asia [16,64]. Part of the explanation for coexistence may rely on life-history trade-offs and abiotic factors [40,65–67] Coexistence between *Ae*. *aegypti* and *Ae*. *albopictus* may be possible due to dry and warm climates that would favor the former and mitigate effects of larval competition via differential mortality of *Ae*. *albopictus* [67]. This hypothesis was reinforced by Camara et al. (2016) [34], observing that intensity of competition at the larval stage may vary seasonally, with harsh effects on development time during warmer Summer. Abiotic factors may also contribute to habitat segregation since urbanized areas tend to be warmer than arborized surrounding areas [68]. Additionally, one force that can impact *Ae*. *aegypti*/*w*MelBr invasion is the asymmetric reproductive interference among mosquitoes, in which male *Ae*. *albopictus* can inseminate and thus sterilize *Ae*. *aegypti* females. The act of reducing the reproductive success of a different species by mating a female of an incompatible species is called satyrization [69–71]. Evidence of satyrization of *Ae*. *aegypti* females seems to be more likely than on *Ae*. *albopictus* females, although still low (less than 5%), biasing the asymmetric nature of cross matings in favor of the latter [72–74]. Therefore, although still not observed in Brazilian sites where *Wolbachia* deployment is ongoing, additional concern would be required if invasion is lagging.

The major force that can affect *Wolbachia* invasion is the population density of wild mosquitoes [75,76]. This concern is even more important if we consider that mosquitoes from other species can lay eggs in the same breeding sites of *Ae*. *aegypti*. Therefore, during *Wolbachia* deployment, infected mosquitoes will lay their eggs in breeding sites already colonized by local mosquitoes, such as uninfected *Ae*. *aegypti* and *Ae*. *albopictus*. Assuming *Ae*. *albopictus* is a better competitor and the presence of *Wolbachia* does not increase mosquito performance at the larval stage, the natural density of *Ae*. *albopictus* may become an additional obstacle to slow invasion. However, we observed a negative growth rate of *Ae*. *aegypti*/*w*MelBr only when *Ae*. *albopictus* frequency was high. In Rio de Janeiro, we selected four neighborhoods with different landscapes and performed adult mosquito collections with BG-Sentinel Traps installed at the peridomestic area of local householders on a weekly basis for 104 consecutive weeks [31]. We observed that the frequency of *Ae*. *albopictus* was lower than 5% in the four sites during the 104 weeks of trapping. In fact, during approximately four consecutive months, no *Ae*. *albopictus* mosquitoes were collected in any trap from any field site (Eliminate Dengue Program). Therefore, *Ae*. *albopictus* is more likely to slow down *Wolbachia* invasion, rather than to stop it. Density-dependent traits can promote strong effects on *Wolbachia* dynamics in *Ae*. *aegypti* field populations [77]. Therefore, an estimation of the population sizes of *Ae*. *aegypti* and other mosquito populations that can occasionally lay eggs in the same breeding sites, such as *Ae*. *albopictus*, *Culex quinquefasciatus* and *Limmatus durhami*, might provide important information on the *Wolbachia* invasion pattern in highly infested field sites [31,76–80].
